# Digital Interventions for Screening and Treating Common Mental Disorders or Symptoms of Common Mental Illness in Adults: Systematic Review and Meta-analysis

**DOI:** 10.2196/20581

**Published:** 2020-09-02

**Authors:** Jacqueline Sin, Gian Galeazzi, Elicia McGregor, Jennifer Collom, Anna Taylor, Barbara Barrett, Vanessa Lawrence, Claire Henderson

**Affiliations:** 1 School of Psychology and Clinical Language Sciences University of Reading Reading United Kingdom; 2 Population Health Research Institute St George's, University of London London United Kingdom; 3 Department of Biomedical, Metabolic, and Neural Sciences University of Modena and Reggio Emilia Modena Italy; 4 Health Services & Population Research Department Institute of Psychiatry, Psychology & Neuroscience King's College London London United Kingdom

**Keywords:** eHealth, mHealth, psychiatric illness, mental disorders, common mental illness, depression, anxiety, self-care

## Abstract

**Background:**

Digital interventions targeting common mental disorders (CMDs) or symptoms of CMDs are growing rapidly and gaining popularity, probably in response to the increased prevalence of CMDs and better awareness of early help-seeking and self-care. However, no previous systematic reviews that focus on these novel interventions were found.

**Objective:**

This systematic review aims to scope entirely web-based interventions that provided screening and signposting for treatment, including self-management strategies, for people with CMDs or subthreshold symptoms. In addition, a meta-analysis was conducted to evaluate the effectiveness of these interventions for mental well-being and mental health outcomes.

**Methods:**

Ten electronic databases including MEDLINE, PsycINFO, and EMBASE were searched from January 1, 1999, to early April 2020. We included randomized controlled trials (RCTs) that evaluated a digital intervention (1) targeting adults with symptoms of CMDs, (2) providing both screening and signposting to other resources including self-care, and (3) delivered entirely through the internet. Intervention characteristics including target population, platform used, key design features, and outcome measure results were extracted and compared. Trial outcome results were included in a meta-analysis on the effectiveness of users’ well-being and mental health outcomes. We also rated the meta-analysis results with the Grading of Recommendations, Assessment, Development, and Evaluations approach to establish the quality of the evidence.

**Results:**

The electronic searches yielded 21 papers describing 16 discrete digital interventions. These interventions were investigated in 19 unique trials including 1 (5%) health economic study. Most studies were conducted in Australia and North America. The targeted populations varied from the general population to allied health professionals. All interventions offered algorithm-driven screening with measures to assess symptom levels and to assign treatment options including automatic web-based psychoeducation, self-care strategies, and signposting to existing services. A meta-analysis of usable trial data showed that digital interventions improved well-being (3 randomized controlled trials [RCTs]; n=1307; standardized mean difference [SMD] 0.40; 95% CI 0.29 to 0.51; I^2^=28%; fixed effect), symptoms of mental illness (6 RCTs; n=992; SMD −0.29; 95% CI −0.49 to −0.09; I^2^=51%; random effects), and work and social functioning (3 RCTs; n=795; SMD −0.16; 95% CI −0.30 to −0.02; I^2^=0%; fixed effect) compared with waitlist or attention control. However, some follow-up data failed to show any sustained effects beyond the post intervention time point. Data on mechanisms of change and cost-effectiveness were also lacking, precluding further analysis.

**Conclusions:**

Digital mental health interventions to assess and signpost people experiencing symptoms of CMDs appear to be acceptable to a sufficient number of people and appear to have enough evidence for effectiveness to warrant further study. We recommend that future studies incorporate economic analysis and process evaluation to assess the mechanisms of action and cost-effectiveness to aid scaling of the implementation.

## Introduction

### Background

There are several reasons to study stand-alone digital technology interventions as the first step in the assessment and management of symptoms of common mental disorders (CMDs). CMDs include different types of depression and anxiety and can cause marked emotional distress and interfere with daily functioning [1,2]. First, access to digital technologies is high in many countries and is increasing in many others [1,2]. Second, mild disorders frequently remit without professional treatment, and, instead, self-management strategies can be learned to ameliorate symptoms and prevent future episodes [3]. Third, there are many digital interventions available for CMDs and their related problems, such as poor sleep [4], and for the promotion of mental well-being such as mindfulness [5]. Some have been subjected to rigorous evaluation [6], whereas others have not been subjected per se but are digital applications of evidence-based therapies such as cognitive behavioral therapy (CBT). Fourth, there is evidence that CMDs are increasing in prevalence in groups such as young women and people aged 55 to 64 years [7], and it is not possible to meet these needs in primary care or specialist mental health service based on current resources and workforce supply [8,9]. Fifth, it should not be assumed that digital interventions are a cost-effective way to meet needs that cannot presently be met by the health workforce. They carry development and maintenance costs, and the work entailed must ensure usability and acceptability. Furthermore, for costs to be offset, the intervention must be accessed by a sufficient number of people who experience benefits above and beyond any other service they may be accessing; ensuring this widespread awareness among people likely to benefit also carries costs [10]. Finally, many people prefer to manage their symptoms without recourse to professional services, often because of a desire for self-reliance but also for reasons such as fear of stigmatization and discrimination and barriers to accessing specialist mental health treatment, for example, because of working long hours, the need for a general practitioner or medical referral, or living in a rural area [11,12].

Our starting point for this review is the development and launch in 2017 of one such digital intervention, *Good Thinking*, for people living and working in London, United Kingdom. Good Thinking provides an initial assessment and signposting to web-based self-guided interventions, including self-care and community-based resources, virtual or otherwise, entirely on the web. This comprises 4 modules: sleep problems, stress, low mood, and anxiety, and includes a self-assessment and signposting to mental health self-management apps, digital therapies (eg, Sleepio for sleep problems [13] or FearFighter—a web-based CBT for social phobia or panic disorder [14]), and conventional services. The apps were approved by *NHS Digital*, the organization in charge of digital services within the UK National Health Service (NHS) using a pre-existing quality control process that included considering the evidence base applied in the digital treatment [15]. The user can choose 1 of these 4 modules and be signposted based on responses to questions on the web-based platform, which can be answered regarding the self or someone they know. Alternatively, the user can use a self-assessment tailored for signposting based on algorithms used for the national telephone helpline, NHS 111.

Good Thinking thus differs from digital therapy delivery, which has been the subject of previous reviews [16-21]. Although these reviews focused on CMDs (such as depression and anxiety disorders [6,18], posttraumatic stress disorder [17,22], and insomnia [16]), they investigated the effectiveness of digital psychotherapies, mostly on CBT provided by health care professionals, although with varying degrees of synchronized or asynchronized guidance delivered on the web. Such interventions tend to follow an assessment conducted by a health professional to validate the diagnosis and include further therapist-delivered psychological interventions using various media. In contrast, Good Thinking exemplifies a new breed of digital mental health interventions that allow users to be in complete control of the process (from access to assessment), intervention (emphasizing self-management), and outcome assessments. These users may have symptoms of CMDs, not necessarily meeting diagnostic or mental health service thresholds or not needing specialist services or conventional therapist-led interventions. Many are primarily interested in seeking digital applications that promote self-care for well-being and signposting to alternative services such as a helpline and peer support forums. As such, this broad range of interventions is likely to be sought by a wide population at a time when many countries are promoting awareness and self-care for mental health, such as Every Mind Matters in England and BeyondBlue in Australia).

To the best of our knowledge, no previous reviews have focused on potentially heterogeneous populations using interventions such as Good Thinking that include a self-assessment to help a web-based user choose their next step in terms of self-management or help-seeking. This review of the interventions and their evaluation will contribute to the development and implementation of more successful applications and hence more effective and sustainable web-based interventions.

### Objectives

This study aims to conduct a comprehensive systematic review of studies of digital mental health services that provide web-based self-assessment and treatment that emphasize on self-care for people with common mental health disorders or subthreshold symptoms. We examined randomized controlled trials (RCTs), the fairest and most robust study design in evaluating the effectiveness of entirely web-based interventions aimed at optimizing mental health–related and intermediate outcomes, including self-care, informal support, and treatment services. We planned to conduct meta-analyses on the (cost-) effectiveness of the interventions on mental well-being and CMD symptom outcomes. Using the research evidence, we also aimed to examine the evidence for the mechanisms of action of such interventions through intermediate or health behavioral change outcomes to mental health outcomes.

## Methods

### Data Sources and Search Strategy

Searches for papers written in English, from January 1, 1999 (when electronic and digital health interventions were first documented) to September 20, 2018, were conducted using MEDLINE (Medical Literature Analysis and Retrieval System Online) and MEDLINE in-process, PsycINFO (Psychological Information), CINAHL (Cumulative Index of Nursing and Allied Health Literature), EMBASE (Excerpta Medica dataBASE), CENTRAL (Cochrane Central Register of Controlled Trials), WoS (Web of Science), ASSIA (Applied Social Sciences Index and Abstracts), DARE (Database of Abstracts of Reviews of Effect), HTA (Health Technology Assessment) published and in-process, and NHS EED (NHS Economic Evaluation Database). Once an initial set of papers from the databases were identified, we performed backward and forward searches in their reference lists and citations of the identified papers for any additional studies. We also contacted the authors of the included papers to retrieve relevant information about their study if this was unclear from the published article. To identify articles not included in our original search, we tracked published protocols of trials identified in 2018 and conducted an update search on MEDLINE, PsycINFO, EMBASE, ASSIA, and WoS for any new publications up to April 9, 2020.

We devised search terms using the population, intervention, comparison, and outcome of interest approach [23]. As the search aimed to be highly sensitive, we employed an initial search strategy that combined search terms for populations (eg, common mental health disorders, adults, depression, and anxiety) and interventions (eg, digital/ ehealth* /mhealth* /web /online /internet adj3 intervention/program*/initiative*/group*). We refined and adapted the search terms used to suit different database search systems. We have published a review protocol in PROSPERO (Prospective Register of Systematic Reviews, CRD42017079085) [24]. The review process followed the PRISMA (Preferred Reporting Items for Systematic Reviews and Meta-Analyses) guidelines [25].

### Study Eligibility and Selection

We included studies that targeted adults aged 18 years, with no upper age limit. According to the UK Adult Psychiatric Morbidity Survey (APMS [7]), CMDs include different types of depression and anxiety and can cause marked emotional distress and interfere with daily functioning, but do not usually affect insight or cognition. Symptoms of CMD include somatic symptoms, fatigue, sleep problems, irritability, worry about physical health, concentration and forgetfulness, depression, generalized worry, anxiety, phobias, panic, compulsions, and obsessions [7]. We also consulted experts in the field to establish whether certain types or symptoms of illnesses, not covered by the APMS definitions, fit the criteria of CMD. Examples include perinatal depression.

We included studies of any digital mental health interventions that aimed to support individuals directly and were fully delivered using web-based information and communication technology (ICT). Facilitation by nondigital resources, such as professionals or lay persons, did not affect study inclusion as far as the intervention was fully delivered using web-based ICT. We specified that intervention contents must include screening or diagnostic assessment and self-care for mental health promotion or symptom management as part of the treatment that can also include information giving, signposting or recommendations, informal support, and pre-existing treatment options. We excluded interventions designed to solely provide assessment or treatment, but not both. To examine the (cost-) effectiveness of the identified interventions, we included only empirical studies using a web-based RCT design for optimal external and internal validity [26] and with intervention recipients’ outcomes reported using validated quantitative measures.

One author (AT, JC, EM, or JS) screened all retrieved items through their titles, abstracts, and then full text. Another author (JS or GG) conducted an independent check on a random 20% sample of all the items at each step and a third author (CH) reviewed a proportion of searches, screening, and study selection. Disagreements were resolved through (1) seeking additional data or clarification from study authors when possible and (2) reviewing during team discussions. All study selection processes were conducted using EndNote software version 8.0 (Clarivate Analytics).

### Outcomes and Measures

For this comprehensive review, we set a range of primary outcomes focusing on participants’ symptoms of CMDs and their related domains. These included symptoms of mental illness, well-being, quality of life, perceived social support, work and social functioning, self-efficacy or coping, and adverse events. Process and/or intermediate outcomes were specified as health behavior change or proxy measures that are conduits to primary outcomes. These included the uptake of recommendations on self-care strategies and increased behavioral activation (such as, goal setting, self-monitoring, and general communication skills) [26]. In addition, we examined data on satisfaction or perceived acceptability of the intervention.

### Data Extraction and Analysis

Relevant extracted data from the included studies were entered into a summary table devised by the review team. We extracted study design and data variables from each included study for further analysis, including sample size, setting, participant characteristics (such as age, gender, diagnosis or symptoms or complaints, and ethnicity), outcome measures, time points, and control condition or comparator. Data on the intervention extracted were as follows: aims, theoretical framework if used and described, content and features, and duration of intervention both in terms of usage hours if specified and the period during which the intervention was undertaken.

Regarding the theoretical framework, we scoped the theoretical basis used by the studies (eg, social cognitive theory, health belief model), the use of theory (eg, theory or predictors used to select recipients for the intervention) in informing intervention design [27], and any behavior change techniques employed by the identified intervention (eg, stress management, goal setting) [28]. We devised a coding system for these factors as they have been established to be particularly effective in promoting intervention uptake and effectiveness [28-30].

Data extracted on the content and features included the following:

1. The modes of delivery, access, and overall approach of the interventions.
2. Web based (ie, eHealth), mobile health (mHealth), or both eHealth and mHealth.
3. With social networking function, no social network, or combined therapy and social networking.
4. Free versus paid versus depending on contract.
5. Treatment options including self-care or management, informal support such as using peer support or community support resources, or signposting to formal or statutory services.

Data analysis started with an overview of study and intervention characteristics, followed by the tabulation of extracted data. All data deemed relevant for each review objective were grouped together and synthesized using a narrative approach. When sufficient homogeneous data were available, we conducted meta-analyses to investigate the effectiveness of treatment using Review Manager (version 5.3, the Cochrane Collaboration). A meta-regression to investigate the significance of identified moderators on treatment effectiveness was considered in the event that 10 studies were included in a meta-analysis [31]. We used a fixed-effects model when <5 studies were included in the meta-analysis and a random-effects model when ≥5 studies were included in the meta-analysis [31]. In addition to conducting overall analyses comparing digital interventions with all comparators pooled together, we also conducted separate comparisons of digital interventions against all inactive controls (eg, waitlist or usual care) and digital interventions against active controls (eg, interventions augmented with a nondigital element such as therapist support via face-to-face or phone contact or attention controls). As the outcomes were measured with different validated scales, we calculated standardized mean difference (SMD) and 95% CI for continuous outcomes and risk ratio and its 95% CI for dichotomous data [32]. Statistical heterogeneity was quantified using the I^2^ statistics in addition to the visual inspection of the forest plots, with I^2^ values >50% interpreted as evidence of substantial levels of heterogeneity [31]. Although some consider SMDs of 0.2, 0.5, and 0.8 as small, medium, and large effects, respectively, the magnitude of these effects alone has been criticized as not having any relationship with their clinical importance [31]. Instead, SMDs should be interpreted within the context of overall quantity and quality of the data included in the meta-analysis (see following sections).

### Assessment of Study and Evidence Quality

We used the integrated criteria for a review of multiple study designs (ICROMS [33]) to assess the quality of the included studies. All studies were assessed for 7 dimensions: clear aims and justification; managing bias in sampling or between groups, in follow-ups, and in other study aspects; analytical rigor; and managing bias in reporting or ethical considerations. Each criterion was evaluated on a 3-point scale (2=criterion met, 1=unclear, 0=criterion not met). The ICROMS minimum score requirement for RCTs, including cluster (ie, 22), was used to rate the trial quality rather than to exclude studies on grounds of quality to retain usable data [33]. In addition, we also used the CONSORT (Consolidated Standards of Reporting Trials) eHealth Checklist (v.1.6.1) [34] to assess trial reporting quality. For health economic studies, we used the Consolidated Health Economic Evaluation Reporting Standards (CHEERS Checklist [35]) to assess specialty study quality. Quality assessment was independently conducted by 2 authors (EM, GG, or JS), and health economic studies were assessed by an expert in the field (BB). In the event of discrepant assessment results, we resolved them for consensus through (1) seeking additional data or clarification from study authors when possible and (2) reviewing during team discussions.

For collective data pooled into meta-analyses, we assessed the quality of the evidence for each analysis using the Grading of Recommendations, Assessment, Development, and Evaluation (GRADE) approach [31,36]. One of the 4 levels—high, moderate, low, or very low—were assigned to the overall quality of evidence for each outcome according to factors including a within-study risk of bias (methodological quality), directness of evidence, heterogeneity, precision of effect estimates, and risk of publication bias.

## Results

**Figure 1:**
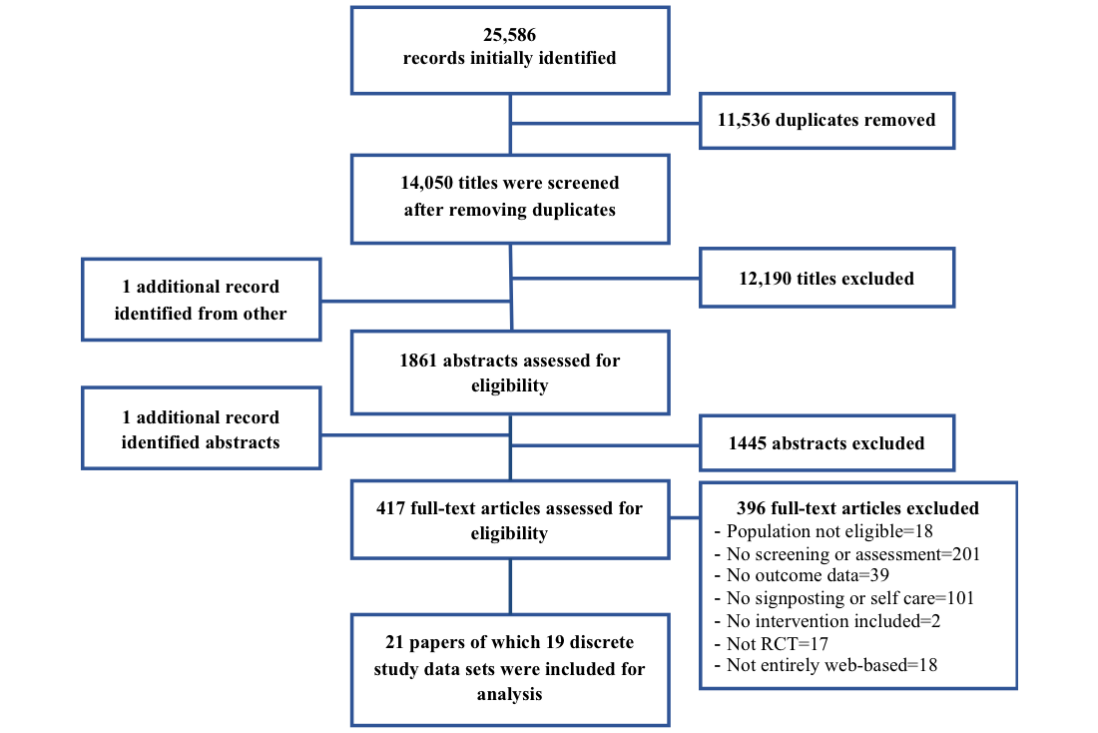
PRISMA (Preferred Reporting Items for Systematic Reviews and Meta-Analyses) flowchart.

The search initially retrieved 25,586 records. A stepwise process of screening titles, abstracts, and full-text papers against our eligibility criteria was used to identify 417 full-text articles for the final screening stage. Of these, 21 papers including 19 discrete study data sets were included [5,37-54]. One RCT paper [55] included partial data from a previous paper that reported on the same tailored eHealth intervention investigated with the same sample in the Netherlands [41]; hence, we only used data extracted from the latter, which also reported trial registration details. Similarly, we included the main paper out of the 2 that reported on the same trial of a digital public mental health program in Hong Kong [43,56]. Results from the search process are shown in Figure 1, and a summary of the included studies is presented in Table 1.

**Table 1:** Summary of the included studies.

Reference; country	Targeted CMD^a^	Intervention approach (n)^b^, gender distribution^c^ (%F/M/other), and age	Comparisons (n), gender distribution^c^ (%F/M/other), and age	Outcomes with validated measures
Batterham [39]; Australia	Depression and anxiety	Web-based assessment with tailored feedback and health information on depression or anxiety, respectively (n=1342, US^d^)	No tailored feedback, just generic advice (n=1431, US)	AHSQ^e^, PHQ-9^f^, GHSQ^g^, AQoL-4D^h^
Batterham [38]; Australia	Depression, anxiety, substance use, and suicidal ideation	FitMindKit, a tailored feedback with 10 core and 8 elective behavior therapy modules based on symptom profile (n=66, 86% F, 14% M, US)	Static FitMindKit—with no tailored feedback (n=62); attention control, a web-based HealthWatch program (n=62, 86% F, 14% M, US)	PHQ-9, GAD-7^i^, PADIS^j^, SOPHS^k^, AUDIT^l^, DUDIT^m^, SIDAS^n^
Billings [40]; United States	Stress, depression, anxiety, and substance abuse	Stress and Mood Management, a web-based multimedia health promotion CBT^o^ program (n=154, 71% F, 29% M, US)	Waitlist control (n=155, 71% F, 29% M, US)	SDS^p^, PNAS^q^, CES-D^r^, BAI^s^, ATSPPPH-SF^t^, SRSQ^u^, WLQ^v^
Chiauzzi [42]; Unites States	Stress, anxiety, and health behaviors	MyStudentBody, a stress-tailored motivational feedback upon completion of 5 web-based questionnaires (n=80, 48% M, 52% F, US)	Control website with no tailoring (n=80); no treatment control (n=80, 48% M, 52% F, US)	PSS-10^w^, HPLP-II^x^, CAS^y^
Eimontas [51]; Lithuania	Adjustment disorder	BADI^z^, a web-based unguided self-help psychological intervention for ICD-11^aa^ adjustment disorder (n=516, 82% F, 18% M, mean age 35 years)	BADI-T^ab^ group—BADI intervention augmented with web-based therapist support (n=561, 82% F, 18% M, mean age 35 years)	ADNM-8^ac^, WHO-5^ad^
Eimontas [50]; Lithuania	Adjustment disorder	BADI, a web-based unguided self-help psychological intervention for ICD-11 adjustment disorder (n=156, 82% F, 18% M, mean age 35 years)	Waitlist control (n=128, 82% F, 18% M, mean age 35 years)	ADNM-8, WHO-5
Farrer [49]; Australia	Depression and anxiety	UVC^ae^, a multicomponent, transdiagnostic web-based mental health program designed for university students (n=102, 78% F, 17% M, 5% other, mean age 22 years)	Waitlist control (n=98, 78% F, 17% M, 5% other, mean age 22 years)	PHQ-9, GAD-7, SOPHS, K10^af^, EURO-HIS 8^ag^, GSE-10^ah^, CSEI^ai^, ATSPPH-SF
Fulmer [48]; Unites States	Depression, anxiety	Tess^aj^, 2 versions of an integrative psychological artificial intelligence chatbox fully automated intervention for 2 weeks with daily check-ins (n=24) or 4 weeks with biweekly check-ins (n=26, 70% F, 29% M, 1% other, mean age 23 years)	Attention control—link to an electronic book on depression (n=24, 70% F, 29% M, 1% other, mean age 23 years)	PHQ-9, GAD-7, PANAS
Haga [53]; Norway	Perinatal depressive symptoms	Mamma Mia, fully automated preventive intervention for perinatal depressive symptoms and usual care (n=678, 100% F, mean age 31 years)	Treatment as usual (up to 14 consultations at well-baby clinic, n=664, 100% F, mean age 31 years)	EPDS^ak^
Ketalaar^al^ [41]; the Netherlands	Stress, functioning, and fatigue	Screening and personalized feedback followed by tailored offer of self-help e-mental health intervention based on symptoms (n=178, 83% F, 17% M, mean age 37 years)	Waitlist control (n=188, 77% F, 23% M, mean age 42 years)	NWFQ^am^, 4DSQ^an^, QEEW^ao^, WAI^ap^, IES^aq^ (Dutch)
Ludtke [47]; Germany	Depression	Be Good to Yourself CBT-based mobile self-help app (n=44, 82% F, 18% M, mean age 41 years)	Waitlist control (n=44, 75% F, 25% M, mean age 45 years)	PHQ-9, Rosenberg Self-Esteem Scale, WHOQOL-BREF^ar^, URICA^as^, CSQ-8^at^
Mak^al^ [43]; Hong Kong	Psychological distress	Living With Heart App providing a mindfulness-based program (n=703) or a self-compassion program (n=705, 73% F, 27% M, mean age 34 years)	Web-based cognitive behavioral psychoeducation program (n=753, 73%, 27% M, mean age 34 years)	WHO-5, K6^au^, MAAS^av^, Self-Compassion Scale
Moberg [5]; United States	Stress, anxiety, and depression	Pacifica, fully automated app for the self-management of stress, anxiety, and depression app (n=253, 74% F, 23% M, 3% other, mean age 30 years)	Waitlist (n=247, 75% F, 23% M, 2% other, mean age 30 years)	DASS-21^aw^, PHQ-8^ax^, GAD-7, GSE-10
Proudfoot [37]; Australia	Depression, anxiety, and stress	myCompass—a fully automated, non–therapist-supported psychological treatment tailored to the user (n=472, 70% F, 30% M, mean age 39 years)	Waitlist (n=230, 70% F, 30% M, mean age 38 years); attention control (n=248, 70% F, 30% M, mean age 40 years)	DASS-21, WSAS^ay^
Querstret [54]; United Kingdom	Stress, depression, and anxiety	Be Mindful Online —a web-based mindfulness-based cognitive therapy course (n=60, 81% F, 19% M, mean age 40 years)	Waitlist (n=58, 81% F, 19% M, mean age 42 years)	Symptom severity
Solomon [52]; Australia	Depression, anxiety, and stress	MyCompass —same as Proudfoot et al [37]. Sample size not applicable because of modeling and simulation used (US)	Antidepressant medication or CBT (US)	Quality-adjusted life years
Stallman [46]; Australia	Psychological distress	My Coping Plan app, offering automated support to building an individualized coping plan (n=28, 91% F, 9% M, mean age 29 years)	Waitlist (n=28, 91% F, 9% M, mean age 29 years)	K10, CI^az^, WHO-5
Viskovich [44]; Australia	Psychological distress	YOLO^ba^ program, a web-based multimedia acceptance and commitment therapy with 4 modules, offered in 3 derivatives: (1) complete 1 module per week but fully flexible (n=40, 75% F, 25% M, mean age 27 years),	(2) to complete the YOLO program in 4 weeks (n=43, 75% F, 25% M, mean age 27 years) and (3) to access a YOLO module 3 days after completion of the previous module (n=47, 75% F, 25% M, mean age 27 years)	DASS-21, MHC-SF^bb^, SCS-SF^bc^, SWLS^bd^, DDQR^be^, AAQ-II^bf^, CFQ^bg^, PVQII^bh^ education values subscale, ELS^bi^, MAAS, SUS^bj^
Viskovich [45]; Australia	Depression, anxiety, and stress	YOLO program—a multimedia acceptance and commitment therapy with 4 modules, as above (n=596, 68% F, 32% M, mean age 27 years)	Waitlist (n=566, 68% F, 32% M, mean age 27 years)	DASS-21, MHC-SF, SCS-SF, SWLS, AAQ-II, CFQ, PVQII education values subscale, ELS, MAAS, SUS

^a^CMD: common mental disorder.

^b^(n): sample size.

^c^Gender distribution: percentage of female, male, or other/unspecified participants.

^d^US: unspecified.

^e^AHSQ: Actual Help Seeking Questionnaire.

^f^PHQ-9: Patient Health Questionnaire-9 items.

^g^GHSQ: General Help Seeking Questionnaire.

^h^AQoL: Assessment of Quality of Life.

^i^GAD-7: Generalized Anxiety Disorder-7.

^j^PADIS: Panic Disorder Screener.

^k^SOPHS: Social Phobia Screener.

^l^AUDIT: Alcohol Use Disorders Identification Test.

^m^DUDIT: Drug Use Disorders Identification Test.

^n^SIDAS: Suicidal Ideation Attribution Scale.

^o^CBT: cognitive behavioral therapy.

^p^SDS: Symptoms of Distress scale.

^q^PANAS: positive and negative affect schedule.

^r^CES-D: Centre for Epidemiologic Studies Depression Scale.

^s^BAI: Beck Anxiety Inventory.

^t^ATSPPPH-SF: Attitudes Towards Seeking Professional Psychological Help Scale-Short Form.

^u^SRSQ: Stress Relief Strategies Questionnaire.

^v^WLQ: Work Limitations Questionnaire.

^w^PSS: Perceived Stress Scale.

^x^HPLP-II: Health-Promoting Lifestyle Profile II.

^y^CAS: College Adjustment Scales.

^z^BADI: Brief Adjustment Disorder Intervention.

^aa^ICD-11: International Classification of Diseases 11th Revision.

^ab^BADI-T: Brief Adjustment Disorder Intervention – Therapist support.

^ac^ADNM-8: Brief Adjustment Disorder New Model Scale.

^ad^WHO-5: World Health Organization well-being index.

^ae^UVC: Uni Virtual Clinic.

^af^K10: Kessler 10 items Psychological Distress Scale.

^ag^EURO-HIS 8: shortened version of the World Health Organization Quality of Life Instrument-Abbreviated Version.

^ah^GSE-10: General Self-Efficacy Scale.

^ai^CSEI: College Self-Efficacy Inventory.

^aj^Tess: name of the intervention.

^ak^EPDS: Edinburgh Postnatal Depression Scale.

^al^Denotes the major publication for the same study sample and data.

^am^NWFQ: Nurses Workforce Functioning Questionnaire.

^an^4DSQ: Four Dimensional Symptoms Questionnaire.

^ao^QEEW: questionnaire on the experience and evaluation of work.

^ap^WAI: Work Ability Index.

^aq^IES: Impact of Event Scale.

^ar^WHOQOL-BREF: World Health Organization Quality of Life Instrument-abbreviated version.

^as^URICA: University of Rhode Island Change Assessment.

^at^CSQ-8: client satisfaction questionnaire.

^au^K6: Kessler 6-Item Psychological Distress Scale.

^av^MAAS: Mindful Attention and Awareness Scale.

^aw^DASS-21: Depression Anxiety and Stress Scales-21.

^ax^PHQ-8: Patient Health Questionnaire-8 items.

^ay^WSAS: Work and Social Adjustment Scale.

^az^CI: coping index.

^ba^YOLO: You Only Live Once.

^bb^MHC-SF: Mental Health Continuum-Short Form.

^bc^SCS-SF: Self-Compassion Scale-Short Form.

^bd^SWLS: Satisfaction with Life Scale.

^be^DDQR: Daily Drinking Questionnaire Revised.

^bf^AAQ-II: Acceptance and Action Questionnaire II.

^bg^CFQ: Cognitive Fusion Questionnaire.

^bh^PVQII: Personal Value Questionnaire II.

^bi^ELS: Engaged Living Scale.

^bj^SUS: System Usability Scale.

### Overview of the Included Studies

Overall, the included studies covered 6223 participants in intervention conditions and 5797 participants in comparison conditions. Nearly half of the studies (8/19, 42%) including a cost-effectiveness study[37-39,44-46,49,52] were conducted in Australia. Four (4/19, 21%) studies were conducted in the United States [5,40,42,48]. The remaining studies took place in Europe, including Lithuania [50,51], the United Kingdom [54], the Netherlands [41], Germany [47], and Norway [53]. Finally, 1/19 (5%) study originated from Hong Kong, China [43].

Studies recruited adults with subclinical or mild symptoms of CMDs among the general population in the community through social media (Facebook and Twitter) advertisements [5,37,38]. Nearly half of the studies aimed at promoting positive well-being and targeted users with some indication of clinical symptoms, including university students [42,44-46,48,49] and the general public who were interested in self-care to promote well-being [5,38,39,43,47,54]. The remaining studies targeted populations with an increased risk of mental health morbidities either because of work-related stress or health conditions. These included nurses and allied health professionals [41], technology company employees [40], and pregnant or postpartum women and their partners to prevent or manage postpartum depression [53]. Very few studies targeted populations with symptoms of CMDs that were above the clinical threshold. The exceptions included studies trialing an electronic mental health treatment for those with mild-to-moderate depression [37] or marked adjustment disorder symptoms [50,51].

Across the included studies, female participants comprised, on average, three-fourth of the overall sample (from 66% to 90%). Participants were largely in their early adulthood (aged 20-30 years). Few studies provided details on other sociodemographic characteristics, beyond age and gender, of the participants, an exception being ethnicity for studies from the United States and Australia. One trial from the United States on university students reported that half of the participants were Asians (50%), outweighing those who were Whites (43%), with only 3% of African Americans or Black participants [48]. The other studies from the United States showed instead a majority of Whites over Asian and Black or African American participants: the percentages were 59%, 18%, and 13%, respectively, in another study on students [42]; 82%, 4%, and 10%, respectively, in a further US app trial [5]; and 65%, 23%, and 7%, respectively, in a web-based stress management program [40]. In Australia, a trial reported that about half (53%) of the participants were Whites, 15% were Asians, 3% were Africans, 0.8% were Aboriginal or Torres Strait Islanders, and a further 17% preferred not to provide ethnicity details [44]. Finally, in an Australian study of a university student virtual clinic, 65% of the participants were Whites, 28% were Asians, 1% were Africans, and 1% were Aboriginal, Torres Strait, and Pacific Islanders [49].

### Intervention Design and Features

Sixteen digital interventions were reported in the 19 included studies: 1 brief adjustment disorder intervention was trialed in 2 RCTs in Lithuania [50,51], a web-based acceptance and commitment therapy intervention was tested in 2 studies in Australia [44,45], and a web-based intervention targeting mild-to-moderate depression was reported in both an effectiveness trial [37] and a health economic study, [52] also in Australia.

In terms of intervention approaches, most offered web-based screening using various validated CMD measures followed by automatically generated (individualized) feedback, including classifying the users’ CMD symptom levels from no risk to high risk. All interventions included offered signposting to relevant services or resources, including self-management strategies such as mood or progress monitoring; relaxation strategies including meditation, mindfulness, and self-compassion; goal setting; journaling; and activating exercises. Some interventions further used the screening results to assign individuals to a relevant web-based mental health treatment pathway using artificial intelligence (AI) algorithms [39,48].

The mode of delivery and design features of the interventions are summarized in Table 2. Most were delivered through a web-based portal allowing users to access it through any device with a web browser [37-39,44,49,50,53,54]. Some were specifically developed and trialed as mobile apps [5,43,46,47]. There was 1 fully AI chat box [48]. All included trials tested digital self-care interventions, often incorporating psychoeducation [39,40,53] and various other psychological intervention modalities. The most commonly employed intervention strategies included mindfulness [5,43,47,50,54], compassion, CBT [5,47,50], acceptance and commitment therapy [44,45], motivational interviewing [48], and positive psychology mobilizing the individual’s strengths [46,48]. Five interventions included an interactive forum where users can exchange discussions with one another [5,38,39,43,46].

**Table 2:** Mode of delivery used by the included interventions.

References	Delivery platform				Social network	Treatment recommendations				Cost		
	App	Computers	Both	Other		Self-care	Informal support	Formal service	Other	Free	Paid	Not stated
Batterham et al [39]	N/A^a^	N/A	X^b^	X	X	X	X	N/A	X	N/A	N/A	X
Batterham et al [38]	N/A	X	N/A	N/A	X	X	X	N/A	X	X	N/A	N/A
Billings et al [40]	N/A	X	N/A	N/A	N/A	X	N/A	X	X	X	N/A	N/A
Chiauzzi et al [42]	N/A	X	N/A	N/A	N/A	X	N/A	N/A	X	N/A	N/A	X
Eimontas et al^c^ [50]	N/A	N/A	X	N/A	N/A	X	N/A	N/A	N/A	X	N/A	N/A
Farrer et al [49]	N/A	N/A	X	N/A	N/A	X	N/A	N/A	X	N/A	N/A	X
Fulmer et al [48]	N/A	N/A	N/A	X	N/A	X	N/A	N/A	X	X	N/A	N/A
Haga et al [53]	N/A	N/A	X	N/A	N/A	X	N/A	N/A	N/A	X	N/A	N/A
Ketelaar et al [41]	N/A	X	N/A	N/A	N/A	X	N/A	N/A	X	N/A	N/A	X
Mak et al [43]	N/A	N/A	X	N/A	X	X	N/A	N/A	X	X	N/A	N/A
Ludtke et al [47]	X	N/A	N/A	N/A	N/A	X	N/A	N/A	X	N/A	N/A	X
Moberg et al [5]	X	N/A	N/A	N/A	X	X	X	N/A	N/A	N/A	N/A	X
Proudfoot et al^c^ [37]	N/A	N/A	X	N/A	N/A	X	N/A	N/A	X	X	N/A	N/A
Querstret et al [54]	N/A	X	N/A	N/A	N/A	X	N/A	N/A	N/A	X	N/A	N/A
Stallman et al [46]	X	N/A	N/A	N/A	X	X	X	X	N/A	X	N/A	N/A
Viskovich et al^c^ [45]	N/A	X	N/A	N/A	N/A	X	N/A	N/A	N/A	X	N/A	N/A

^a^N/A: not applicable.

^b^X: indicated feature.

^c^Data from Eimontas 2018 [50], Proudfoot 2013 [37], and Viskovich 2019 [45] used for intervention description here.

Limited details of the digital intervention designs and ICT features were reported. An explicit theoretical basis underpinning the design and delivery integrating algorithm and web-based behavioral change techniques was generally lacking. Across studies, only a few web-based behavioral change techniques were explicitly adopted by the interventions, including provision of feedback on performance [39,51], goal setting [46], and prompts for self-monitoring of behavior and progress [37,43,49]. Intervention duration and intensity varied widely across studies, with most interventions lasting 4 weeks [43,44,46], a few lasting 3 months [38,39,41], and the longest lasting 11 months [53]. Most interventions did not stipulate the minimum usage requirement and recommended that the users use the intervention as preferred [38]. Some interventions had a set number of modules to be undertaken over a set time frame. However, these did not necessarily translate into minimum usage requirement, intervention duration, or intensity [37,38,53].

### Study Design and Outcome Measures

All but one of the included studies used an individual-level RCT design. Only 1 study used a cluster RCT design at a ward level where nurses and allied health professionals were allocated according to their work base within a hospital in the Netherlands [41]. All studies examined digital intervention effectiveness, with 1 including a health economic modeling study comparing cost-effectiveness of the digital intervention with antidepressant medication (as treatment as usual) or CBT for mild-to-moderate depression in Australia [37,52]. The comparison conditions used in the included RCTs were grouped into (1) inactive controls and (2) active controls. The former includes usual care delivered using a conventional medium [53] or waitlist controls [5,37,40,41,45-47,49,50,54]. The latter comprises attention controls (eg, static websites with information or an electronic book [37-39,48]). One trial included 3 arms, comparing the digital intervention with both attention and waitlist controls [37]; we used such data in separate analyses. Two 3-arm trials compared 3 different formats of the same digital intervention head-to-head with no other comparison groups comprising nondigital elements [43,44]. No usable comparison data could be extracted for analyses. Data from 1 trial that compared an entirely web-based self-care intervention for university students with a version of the intervention augmented with therapist input also delivered through its web-based platform was not usable in the analysis [51].

All trials that investigated the effectiveness of digital interventions used outcome measures of symptoms of mental illness, including stress, anxiety, depression, and general distress. Three studies measured well-being [45,46,50] and only 1 study measured quality of life at post intervention and 3-month follow-up, respectively [39,47]. Help-seeking attitude [40] and service use [39] were each measured by 1 study at each time point. Work or general functioning was assessed in 3 studies [37,40,41]. Two studies reported coping as an outcome, with each focused on overall coping [46] or negative coping [40]. Satisfaction with intervention, if assessed, focused only on the intervention group participants and the measures or tools used were often unvalidated or devised by the study teams on an ad hoc basis [44,45,50].

In terms of intermediate outcomes, 1 study measured knowledge of symptoms of CMD, prevention, and treatment [40]. Use of health-promoting behaviors was covered in only 1 study [42], although many reported therapy-specific measures to assess engagement with therapy approaches (eg, compassion, cognitive flexibility, willingness to change). Although behavior change techniques, most often goal setting, prompts for self-monitoring, or action planning, have been reported to form part of the intervention design [37,43,46], no data on uptake of recommendations or behavioral activation outcomes were available if measured.

### Overall Study Quality

Our evaluation of the study quality and the comparison of the global ICROMS score of each study against the ICROMS minimal score requirement is presented in Table 3. The ICROMS global quality scores ranged from 14 to 29; 6 (33%) trials were rated below the minimum score of 22. Although the RCTs were published relatively recently, some did not fully adhere to the CONSORT or CONSORT-eHealth checklist [40]. Many of the RCTs did not publish their protocols or prospectively register the study on trial databases to provide details on the intervention design and required minimum intervention exposure (ie, per-protocol use) or state a priori primary outcomes [42,45,46]. Although randomization and allocation using a computerized or web-based system were often cited, details on the randomization sequence generation and allocation concealment were often minimal if at all reported [5,43,44,47,50,51]. Given that waitlist control or usual care was most commonly used as the comparator, it was not feasible to blind the participants, although there were few exceptions [49,54]. Although outcome data collection using web-based questionnaires with the participants directly reduced bias in assessment, limited considerations were conveyed to establish whether the researchers or trial statisticians who conducted the data analysis were blinded to group allocation [5,43,44,47,50,51]. Nonetheless, the most significant quality issue identified here concerns retention and completion rates in digital health intervention trials. An intention-to-treat analysis was not always used, and there was a lack of available data for noncompleters [40,41,44,45,50,51]; these quality issues might bias the study results and overall evidence. Another area of potential bias lies in reporting or ethical considerations, as not all studies reported their funding sources and conflicts of interest. Furthermore, some trialists reported a digital intervention produced by commercial enterprises in which they had a financial interest [5,42,48].

We rated the quality of the health economic study [52] as satisfactory according to CHEERS [35]. The paper addressed 18 of the 24 (67%) CHEERS quality criteria, including clear reporting of method, analysis, results, and discussions. Four checklist criteria were deemed irrelevant in this study (eg, not a single study-based economic evaluation and hence no such study parameters). Quality criteria that were not addressed were discount rates used for costs and outcomes (if any) and justification of the choice of model used.

**Table 3:** Quality assessment of the included studies using integrated criteria for review of multiple study designs (ICROMS).

References (first author only)	Study design^a^	Aims and justifications	Sequence generation and allocation concealment	Outcome measures and blinding	Follow-up	Other study aspects	Analytical rigor	Other considerations	Global quality score
Batterham et al [38]	RCT^b^	2	3	3	5	2	2	11	28^c^
Batterham et al [39]	RCT	2	4	4	6	2	2	8	28^c^
Billings et al [40]	RCT	0	2	1	3	2	1	5	14^d^
Chiauzzi et al [42]	RCT	2	4	2	5	2	2	7	24^c^
Eimontas et al [50]	RCT	1	1	2	5	2	1	7	19^d^
Eimontas et al [51]	RCT	2	1	4	5	2	1	8	23^c^
Farrer et al [49]	RCT	2	4	4	5	2	2	9	28^c^
Fulmer et al [48]	RCT	2	2	4	4	2	1	4	19^d^
Haga et al [53]	RCT	2	3	4	6	2	2	9	28^c^
Ketelaar et al [41]	cRCT^e^	2	4	2	6	2	0	6	22^c^
Ludtke et al [47]	RCT	1	2	4	6	2	2	7	24^c^
Mak et al [43]	RCT	2	2	4	6	2	2	8	26^c^
Moberg et al [5]	RCT	2	1	4	4	2	1	5	19^d^
Proudfoot et al [37]	RCT	2	3	2	5	2	2	8	24^c^
Querstret et al [54]	RCT	2	4	6	6	2	2	7	29^c^
Stallman [46]	RCT	2	4	4	5	2	1	7	25^c^
Viskovich and Pakenham [44]	RCT	1	1	4	4	1	1	6	18^d^
Viskovich and Pakenham [45]	RCT	2	2	3	4	1	1	6	19^d^

^a^ICROMS minimal score requirement for (cluster) randomized controlled trial=22.

^b^RCT: randomized controlled trial.

^c^Comparison against minimal score requirement: above requirement.

^d^Comparison against minimal score requirement: below requirement.

^e^cRCT: cluster randomized controlled trial.

### Effectiveness

**Figure 2:**
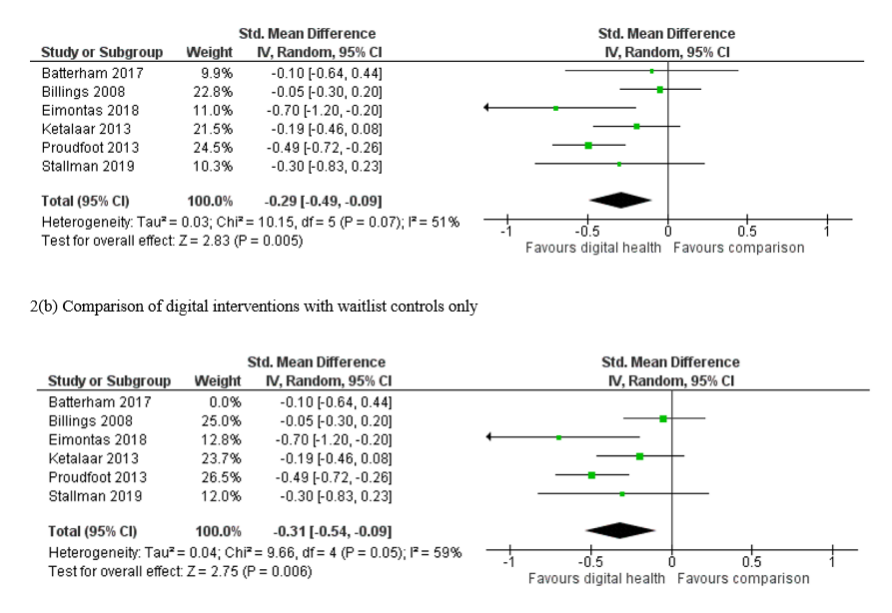
Meta-analysis on outcome of mental illness symptoms 2(a) Comparison of digital interventions with any comparators using all available data.

Six RCTs [37,38,40,41,46,50] reported outcomes using measures of mental illness symptoms (as a composite measure encompassing depression, anxiety, and distress or psychological distress) at the end of the intervention use. These studies examined the effectiveness of tailored digital interventions compared with waitlist controls [37,40,41,46,50] or attention controls [38]. The meta-analysis including these 6 studies showed an overall significant small effect of digital interventions compared with controls in reducing the symptoms of mental illness (6 RCTs; n=992; SMD **−**0.29; 95% CI **−**0.49 to **−**0.09; I^2^=51%; random effects; GRADE quality of evidence=moderate). Comparing digital interventions with waitlist controls only using data from 5 trials led to a similar result favoring digital interventions (5 RCTs; n=939; SMD **−**0.31; 95% CI **−**0.54 to **−**0.09; I^2^=59%; random effects; GRADE quality of evidence=low). Only 2 trials provided data for comparing digital interventions with attention controls [37,38]. The meta-analysis including these data still yielded a significant result favoring digital intervention (2 RCTs; n=374; SMD **−**0.31; 95% CI **−**0.52 to **−**0.10; I^2^=0%; fixed effect; GRADE quality of evidence=very low). Figure 2 shows the meta-analyses on the outcome of symptoms of mental illness.

**Figure 3:**
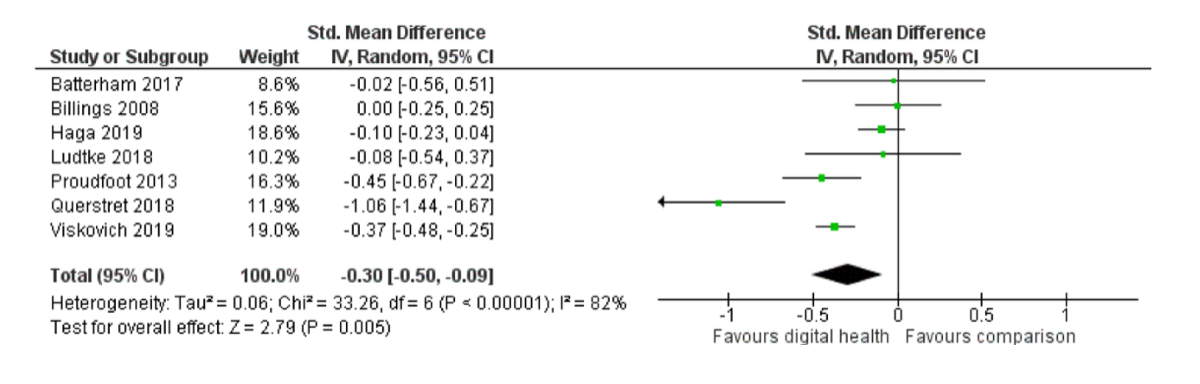
Meta-analysis on outcome of depressive symptoms.

Seven studies measured participants’ depressive symptoms [37,38,40], comparing digital interventions with inactive controls [37,40,45,47,53,54] or attention controls [38]. Digital interventions showed a small but significant positive effect over any comparison (7 RCTs; n=2824; SMD **−**0.30; 95% CI **−**0.50 to **−**0.09; I^2^=82%; random effects; GRADE quality of evidence=low). Heterogeneity of this meta-analysis was high: 3 were European studies, including 1 focusing on postnatal depression in new mothers through a year-long intervention across the perinatal period [53]; 3 were conducted in Australia, comprising nearly half of the total participants in this analysis; and the remainder was conducted in the United States. Figure 3 shows the meta-analysis of depressive symptoms.

**Figure 4:**
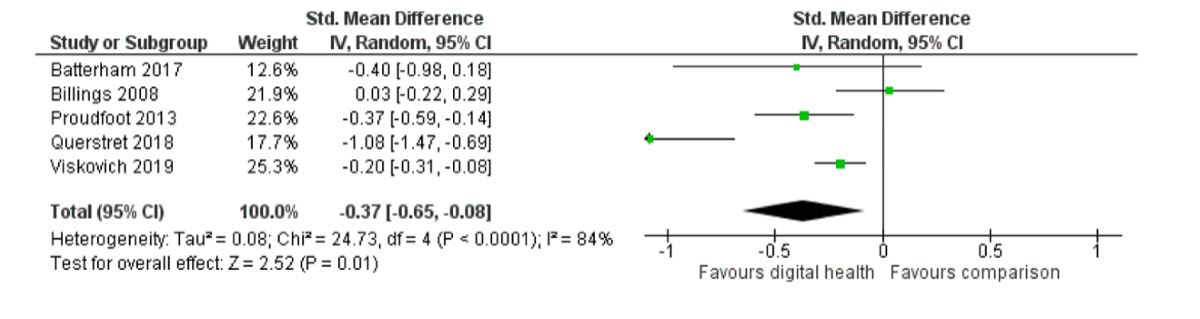
Meta-analysis on outcome of anxiety symptoms.

**Figure 5:**
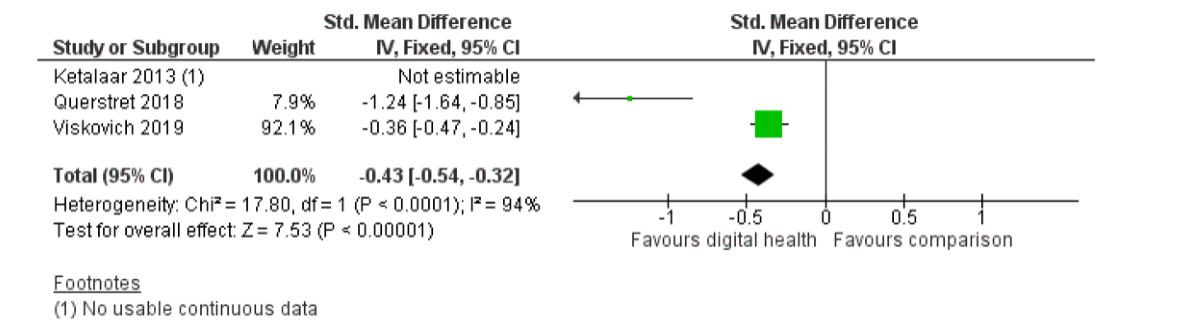
Meta-analysis on outcome of stress symptoms.

A meta-analysis of participants’ anxiety symptoms from 5 studies produced similar positive results favoring digital interventions over inactive or attention controls (5 RCTs; n=1893; SMD **−**0.37; 95% CI **−**0.65 to **−**0.08; I^2^=84%; random effects; GRADE quality of evidence=low). The high heterogeneity is likely because of diverse intervention, population, and methodological factors [37,38,40,45,54]. Figure 4 shows the meta-analysis of the anxiety symptoms. Three studies reported stress outcomes, but only data from 2 of these were used in the meta-analysis [41,45,54]. The analysis showed a significant positive effect over waitlist controls (2 RCTs; n=1280; SMD **−**0.43; 95% CI **−**0.54 to **−**0.32; I^2^=94%; fixed effect; GRADE quality of evidence=very low). Of note, the heterogeneity of these 2 studies was high: 1 trialed a web-based mindfulness CBT for UK workers [54] and the other investigated a web-based acceptance and commitment therapy for university students in Australia [45]. Figure 5 shows the meta-analysis on stress outcomes.

**Figure 6:**
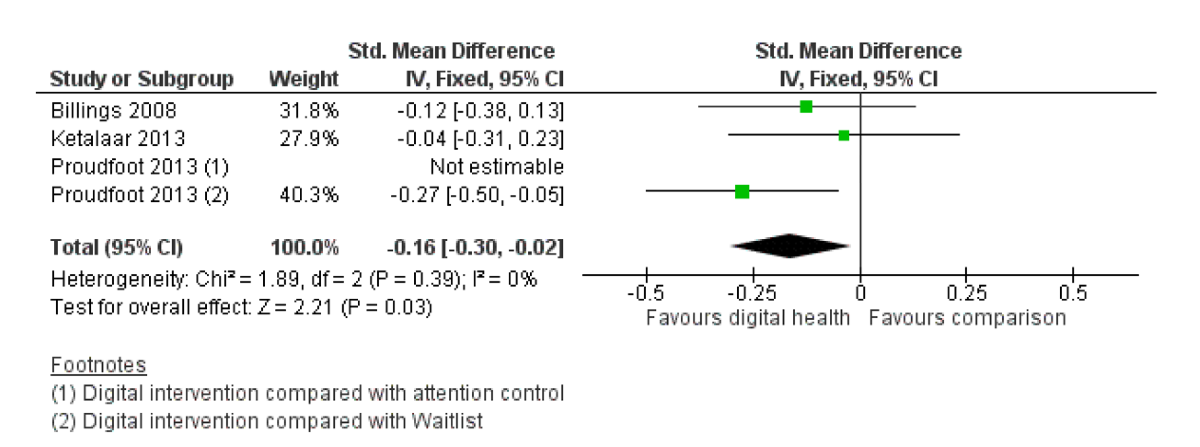
Meta-analysis on outcome of work and social functioning comparing digital interventions with inactive controls.

In terms of work and social functioning outcomes, 3 studies compared digital interventions with inactive controls [37,40,41], whereas 1 study included a second control group using attention controls [37]. Results comparing digital interventions with any comparators were equivocal across groups (3 RCTs; n=792; SMD **−**0.13; 95% CI **−**0.27 to 0.01; I^2^=0%; fixed effect; GRADE quality of evidence=low). However, when comparing digital interventions with inactive controls only, digital interventions showed a significant although small effect over waitlist controls (3 RCTs; n=795; SMD **−**0.16; 95% CI **−**0.30 to **−**0.02; I^2^=0%; fixed effect; GRADE quality of evidence=low). Figure 6 provides the meta-analysis on work and social functioning outcomes.

**Figure 7:**
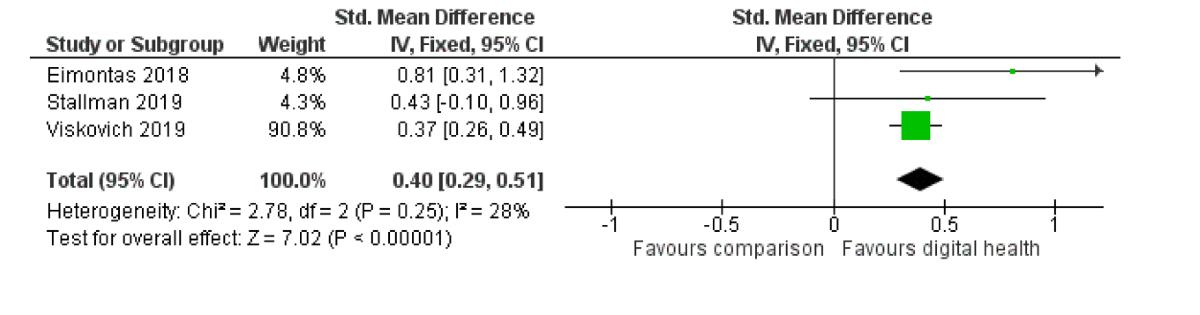
Meta-analysis on outcome of wellbeing comparing digital interventions with inactive controls.

Three studies examined the effectiveness of digital interventions on well-being [45,46,50]; digital interventions delivered as web-based CBT, acceptance, and commitment therapy or mobile app showed a significant positive effect over waitlist controls (3 RCTs; n=1307; SMD 0.40; 95% CI 0.29 to 0.51; I^2^=28%; fixed effect; GRADE quality of evidence=low). It is worth noting that this result was weighted heavily by 1 study conducted in Australia including >1100 university students [45]. Figure 7 provides the meta-analysis on the well-being outcome. Only 1 study measured participants’ quality of life as an outcome when comparing digital intervention with waitlist control [47].

### Follow-Up Outcome Data

Follow-up data (beyond 3 months) were limited. Four studies [37-39,41] provided data, with 1 study delivering 2 active interventions that focused on depression or anxiety management compared with attention controls [39]. Meta-analyses using the available 3-month follow-up data revealed no significant differences in mental health, work, and social functioning outcomes between digital interventions and controls, active or inactive. Table 4 provides a summary of the meta-analysis results using the fixed effect model.

**Table 4:** Summary of meta-analyses on the 3-month follow-up outcome measures.

Outcome measures	Studies, n	Sample, N (n/n)^a^	SMD^b^	95% CI	I^2c^ (%)
Symptoms of mental illness	3	521 (194/327)	−0.12	−0.30 to 0.05	1
Depression	3	1209 (509/700)	−0.04	−0.15 to 0.08	0
Anxiety	3	1044 (431/613)	−0.20	−0.87 to 0.47	0
Work and social functioning	2	476 (171/305)	−0.13	−0.32 to 0.06	0

^a^Total number of participants included in the analysis (number of participants in digital interventions or number of participants in comparator groups).

^b^SMD: standardized mean difference.

^c^I^2^: Statistical heterogeneity.

### Health Economic Outcomes

No RCTs included a cost-effectiveness evaluation. One Australian RCT on a digital intervention, myCompass, designed to treat mild-to-moderate depression in the general population [37], was used as the basis of a decision analytic model [52]. The model employed a cost-utility framework to compare the costs of myCompass with each treatment as usual (antidepressant treatment and face-to-face CBT). The results of the model suggested that the myCompass intervention provided the highest net monetary benefit, and the authors concluded that digital interventions could provide a cost-effective route to treatment as part of a stepped care model [52].

### Intermediate or Process Outcomes

There were no usable data available from RCTs on any of our prespecified intermediate or process outcomes (eg, uptake of self-care or informal support, health behavior change), precluding analysis on such outcomes in its own right or meta-regression on any association between intermediate and health outcomes. Some studies reported therapy-specific mediating measures, such as willingness to change measure in a CBT-based mobile app [47], self-compassion, or 5-facet mindfulness questionnaires in third-wave web-based CBTs [45,54]. These fell short of health behavior change outcomes and were therapy specific; therefore, we considered it inappropriate to compare such outcomes across studies.

### Perceived Acceptability of Interventions

If reported, study findings on satisfaction were collated via self-devised measures or unvalidated survey post intervention use, lacking corroboration from validated outcome data and comparison with the control groups or any other interventions. No analysis of this outcome was feasible.

## Discussion

### Principal Findings

This comprehensive review included 18 RCTs and 1 health economic study on 16 interventions to examine the effectiveness of digital interventions that provided both initial assessment and treatment that emphasize self-care, using a web-based medium entirely. Fourteen of the included trials were only published in the last 5 years, suggesting that despite the popularity of digital mental health interventions, rigorous research undertaken in this field is still emerging.

Our review identified some evidence to support the effectiveness of digital interventions in promoting well-being among university students [45,46,50] and in reducing symptoms of CMDs, depression, anxiety, stress, and promoting social and work functioning. These positive results on the symptoms of CMDs came from studies on nonclinical young adult samples (aged between their early 20s and 30s) among the general population with mild baseline symptoms [37,38,41,45,46,50,54]. It is highly plausible that the recruited study samples included a high proportion of people who had a low intensity of CMD symptoms that might not meet the threshold of clinical caseness or the need for conventional mental health interventions delivered by clinicians (eg, CBT or counseling). Uptake of interventions showed that the majority of the participants had a White background, with Asians being the second most frequent group reported and Blacks being third. Unfortunately, information about ethnicity of the participants was available for only 6 studies [5,40,42,44,48,49], limiting the analysis of plausible cultural determinants of digital health performance. Similar to conventional trials on psychological interventions delivered face-to-face, two-third of the study participants were female [16-18]. Furthermore, some of the included studies were designed primarily as a mental health promotion or preventative intervention, for example, for college students and new mothers [46,49,53]. Despite this aim, there was, in general, a lack of focus on positive psychological outcomes such as well-being or quality of life. Furthermore, there may be a ceiling effect with respect to the population means at baseline or study entry, leaving little room for improvement in the outcomes.

Most of the interventions examined were designed to be accessed and used autonomously by the users [5,37,43,48,50]. Commonly, users were advised to use the intervention flexibly to suit their own preference as much or as often as necessary or desired, although encouraged to make full use of the intervention elements (eg, forum, exercises, and monitoring) and content. A small proportion of interventions, however, guided their users through core content through a specific sequence (eg, to complete 4 modules in a predetermined order [38,47]) or over a specific timeframe (eg, 1 module a week or at a certain time point, such as 3 weeks after giving birth [53]). Although web-based recruitment across studies was largely successful, retention and completion rates reported across trials are concerning. With a couple of trials achieving retention rates ≥80% (eg, [46,48]) as exceptions, attrition rates range from 27% in an Australian trial of a digital depression and anxiety intervention [37] to 78% in a mobile app trial [5,43] and 87% in a web-based intervention [50] post intervention. Attrition at short-term follow-up is equally high (eg, 83% at 3-month follow-up [39]), whereas most of the included studies did not report follow-up beyond the immediate post-intervention time point. Furthermore, the low usage or adherence rate across trials was often cited to account partly for the equivocal results across groups [5,43,50], raising the possibility that no effect was because of low or no minimally sufficient treatment dosage. The low use of digital interventions also prompts doubts over the value of the automatic reminders (as emails or SMS, or mobile app prompts) integral to digital intervention design and delivery in the entirely self-guided treatment. Our review demonstrated that although many interventions sent automatic daily or weekly reminders or prompts to the participants directly, they were not responding accordingly. These issues, although consistent with the inherent challenges of conducting digital intervention trials [57,58], remain critical to be resolved. For any digital interventions to effect meaningful changes in their users, developers need to articulate the essential intervention elements and the required intervention exposure or usage to achieve that as a crucial part of the intervention design [59]. Most importantly, it is essential for digital interventions to optimize their engagement and facilitation strategies to ensure users get the intended benefits of the intervention when enjoying their autonomy in pursuing individualized treatment. The effects of reminders and prompts functions and indeed other communication strategies afforded by digital interventions should be carefully investigated to inform both the intervention and the study designs.

In addition to the paucity of research in the growing field of digital health interventions, we note some limitations in the included studies and the data they reported. Although all interventions examined included a self-assessment component, we found no data pertaining to the effectiveness or efficiency of the assessment function independent of the overall intervention, including their treatment component. Thus, no conclusions could be drawn on the impact of assessment on the users’ initial engagement with the intervention, subsequent signposting based on AI, or the users’ mental health outcomes. Follow-up data were sparse, limiting analysis on outcomes beyond the 3-month follow-up. The lack of reporting on intermediate outcomes and process evaluation data (if used) precluded any analysis to convey how digital interventions might work to instill health outcome changes [26,27]. Although some behavior change techniques were incorporated as intervention design (eg, prompts for self-monitoring or goal setting), data on the target health behavior change outcomes were generally not collected or not reported. Although it is often argued that digital interventions carry with them the benefit to be expanded and delivered to whole populations at a relatively low cost, no data were available regarding estimated cost-effectiveness and only 1 paper included economic modeling [52]. This, coupled with the unclear intervention design description, limits the generalizability of the results and the scope of replication and wider implementation.

### Limitations

This review has several limitations. First, we are mindful that our results are synthesized from studies that reported different interventions targeting a wide range of populations, ranging from those promoting positive mental health to others identifying and treating mild-to-moderate depression. The results therefore fall short of identifying specific intervention designs (eg, with specific ICT features), which may be particularly effective for specific populations or groups with CMDs (symptoms). This approach also, in part, accounted for the high heterogeneity observed in the results of the meta-analysis. Second, given the limited amount of usable data included in the analysis, especially in the follow-up timepoint, we conducted a meta-analysis using the fixed-effect model on endpoint mean score whenever <5 study data sets were available. Although the fixed-effect model is deemed most suitable for a meta-analysis including ≤5 studies, this approach is inferior in taking baseline measurements into consideration, which is particularly important in small trials [32]. We therefore downgraded the GRADE quality ratings accordingly [31].

### Implications for Research and Practice

Although the results from the studies reviewed appear promising, they are limited in terms of generalizability to digital interventions scaled up for use by whole populations. For example, a key implication of the results for both research and practice is the need for economic evaluation of digital mental health services for general population samples [10,60]. Although the usual trial methods for cost-effectiveness evaluation would be informative, economic evaluation of the scaling up of digital interventions to whole populations is also important, as a key consideration for economic evaluation is the potential range of reach of digital services. Whereas widespread awareness and usage of a digital service may increase its cost-effectiveness, creating that awareness also has to be done in a cost-efficient manner. People who use non–digital health or other services can be informed of a digital service at these services, whereas those who only do so rarely or when in crisis but may benefit must be reached by other means. Given that the interventions are web based, the most obvious approach is to use social media advertising in response to mental health–related search terms [9,61]. An economic evaluation of scaling up requires a study of the costs to create awareness of the service and modeling methods using the usage data from the service. Such models must take into account as 1 of their assumptions the additional use of other services, both digital and nondigital, by some users. This is likely to vary as usage increases: as more people take up a digital intervention, the proportions that were previously using something else (and what that was) versus nothing is likely to change; similarly, the intervention’s cost-effectiveness is likely to vary by demographic and clinical groups, which again changes with increasing levels of use.

Outside of a research or practice setting, the extent to which a digital service is trusted is important in addition to its usability [26,28,58]. One implication is the need for research into aspects that affect this trust and how this varies within the general population, for example, the need to use personal information to register before using the intervention and the use of health services or government logos [62].

### Conclusions

Digital mental health interventions to assess and signpost people experiencing symptoms of CMDs appear to be acceptable to a sufficient number of people and have enough evidence for effectiveness to warrant further studies. We recommend that future studies incorporate economic analysis; much of the work in this area appears to rest on the untested assumption that digital interventions are cost-effective by their nature. We also suggest clarification of the theoretical models for interventions. Many apply therapies, such as CBT and psychoeducation, to a sample with milder problems than those presently receiving them and state their aims as including both reduction in symptoms and promotion of mental health. However, positive mental health outcomes such as mental well-being, self-esteem, self-efficacy, coping skills, or resilience are rarely used. This may obscure their effectiveness in the target population. Finally, process evaluation to assess implementation and mechanisms of action are needed to understand the outcomes reported, if needed, in a separate publication.

## Abbreviations

AI: artificial intelligence
APMS: Adult Psychiatric Morbidity Survey
ASSIA: Applied Social Sciences Index and Abstracts
CBT: cognitive behavioral therapy
CHEERS: Consolidated Health Economic Evaluation Reporting Standards
CMD: common mental disorder
CONSORT: Consolidated Standards of Reporting Trials
EMBASE: Excerpta Medica dataBASE
GRADE: Grading of Recommendations, Assessment, Development, and Evaluation
ICROMS: integrated criteria for review of multiple study designs
ICT: information and communication technology
MEDLINE: Medical Literature Analysis and Retrieval System Online
NHS: National Health Service
PsycINFO: Psychological Information
RCT: randomized controlled trial
SMD: standardized mean difference
WoS: Web of Science
